# Refractory Delusional Parasitosis in a 70-year-old Woman

**DOI:** 10.7759/cureus.3120

**Published:** 2018-08-08

**Authors:** Anthony D'Auria, Taylor Wiseman, Anjali Varghese, Eduardo D Espiridion

**Affiliations:** 1 Medical Student, West Virginia School of Osteopathic Medicine, Lewisburg, USA; 2 Psychiatry, West Virginia School of Osteopathic Medicine, Martinsburg, USA

**Keywords:** parasitosis, delsuional

## Abstract

This is a case report of delusional parasitosis in a 70-year-old woman. Delusional parasitosis, or delusional infestation, is a rare disorder typified by the false belief that an individual is infected with insects or parasites. Management focuses on developing a strong, caring relationship with the patient. It is widely debated whether health care providers should agree or disagree with the patient’s delusions. Due to the rarity of this disorder, our hope in presenting this incidence is to motivate more research for treatment.

## Introduction

Delusional parasitosis, or delusional infestation, is a rare disorder typified by the false belief that an individual is infected with insects or parasites. The infestation may manifest on the patient’s skin and/or possessions [1]. In short, the pathogenesis for the disorder is unknown; however, the Diagnostic and Statistical Manual of Mental Disorders, Fifth Edition (DSM-5) classifies it as a delusional disorder, somatic subtype [2]. The prevalence of delusional disorders in their entirety is about 0.05% to 0.1%, making the parasitosis subtype a fraction of that percentage [3-5]. The most common comorbid condition is depression but anxiety is also commonly associated [6].

Although a mental disorder, patients with delusional parasitosis are unaware their symptoms are delusions, and are therefore reluctant to see a psychiatrist. As a result, the disorder is likely underdiagnosed, and dermatologists may be seeing the majority of these patients. Given that assumption, it is still exceedingly rare amongst dermatologists as well, and estimates have been made that a dermatologist may see only one patient with delusional parasitosis every seven years [7].

The phenomenon of delusional parasitosis is most often seen as a symptom of drug intoxication, specifically amphetamine and cocaine [8]. Delusional parasitosis can also be a primary disorder, or occur secondarily to other medical illnesses, or following mental anguish [2,7].

## Case presentation

A 70-year-old Caucasian female presented to an outpatient Partial Psychiatric Hospital Program (PPHP) after an episode where the patient was reported missing, and subsequently found by the police hiding under a tree with minimal clothing in freezing cold temperatures. The patient’s brother and sister-in-law, whom she resides with, were unable to locate her, and reported her missing to the police. Upon discovery by the police, the patient agreed to attend the outpatient PPHP. Upon admission, the patient explained that she ran away from her home due to feelings of guilt and “feeling like a burden to her family.” She then revealed that her feelings of guilt are primarily regarding her perceived parasite infestation, which she states has plagued her for decades. The patient states there are “little white bugs crawling in and out of my skin.” She further explained that they are difficult for her to capture because they dive deep into her skin. The patient expressed fear that her family members will also become infected, prompting her recent episode of escaping her home. She also mentioned feeling embarrassed about the issue, particularly because she is from a rural town and feels everyone will know.

The patient admits she has seen several healthcare providers including her primary care provider, parasitologists, and dermatologists, all reporting negative findings. When explaining this, the patient became very agitated and repeated, “I’m not crazy, but no one believes me.” Additional past medical history was benign other than the patient revealing she underwent an abortion at the age of 35. She does not link the parasite infestation with this event, but does express guilt over this decision.

The patient denied a history of physical or sexual abuse. She denied alcohol or drug abuse. She has never been married and has no children. Upon exam, the patient looked appropriate and stated her age. She maintained eye contact and spoke with coherence. The patient was anxious. Immediate retention and recall, recent memory, remote memory and fund of knowledge appeared to be fair. Insight and judgment were poor. The patient was not impulsive. The patient's current medications include Zyprexa 2.5 mg per os (PO) at bedtime and Zoloft 75 mg PO daily. A toxicology screening of the patient was negative.

From reviewing the medical records, it was elucidated that the patient was admitted to the Behavioral Health Unit two times previously with the same complaint of parasite infestation. The patient could not pinpoint when the supposed infestation began, but does state that it had been many years, possibly 15 years.

When questioned why the patient was at the PPHP, she states that after she was found by the police, she was taken to the Emergency Department (ED) and was medically cleared, but her family demanded she attend the outpatient PPHP because of her delusions. We were also able to ascertain that a computed tomography (CT) scan was taken in the ED, which demonstrated microvascular ischemic changes and cortical atrophy.

On completion of the interview, the patient was observed in group therapy, and a decision was made that the patient was not a good candidate for the PPHP, as her delusions were too severe and becoming disruptive to the group. At the request of her family, she was then transferred to a free-standing private psychiatric hospital for further evaluation and treatment. She currently remains there and is undergoing treatment.

## Discussion

Given the patient’s lack of physical exam findings, it is likely the patient has primary delusional disorder, somatic subtype, with the delusion of infestation as diagnosed by DSM-5 criteria. This case highlights the refractory nature of this patient’s delusion.

The DSM-5 outlines five criteria for the diagnosis of primary delusional infestation. First, the patient has presented with the delusion for longer than one month as noted by her continued admissions for the same chief complaint over the past few years. Second, she has never been diagnosed with schizophrenia. Third, as demonstrated in her physical exam findings, her functioning is not markedly impaired, nor would her behavior be considered bizarre or odd. Fourth, the disturbance is not attributable to substance use or other comorbid conditions which is supported by the negative toxicology screen. The fifth and final criteria for primary delusional infestation states that if manic or major depressive episodes have occured, they have been brief in relation to the delusions [2]. The patient’s past medical history and interview did not reveal any signs of depression.

The patient’s CT scan demonstrating mild ischemic changes and cortical atrophy could be an etiology for delusions, as white matter changes can result in cognitive decline [9]. However, we feel this is not the case for this patient despite her positive CT scan findings given the length of time the patient has been experiencing the delusions in relation to these age-related changes.

Given that the patient meets the five DSM-5 criteria listed above, her repeated admissions and negative findings from various providers including dermatologists, it is our opinion that this patient has primary delusional disorder, somatic subtype.

The mainstay of treatment for a patient carrying this diagnosis is developing a strong, caring relationship, as often times these patients have often have had inadequate treatment from their previous providers who dismiss their claims of infestation [10]. It is widely debated whether health care providers should agree or disagree with the patient’s delusions [11]. Several authors propose a passive approach, using phrases such as “I don’t see the parasites,” rather than overtly saying “you do not have parasites" in order to build the patient's trust [11-12].

In addition to psychotherapy, antipsychotics are the first line medication. Meta-analysis have not found evidence that any particular antipsychotic is more effective than another [13]. We recommend using an antipsychotic that has a favorable side effect profile for the patient.

One etiology of delusional disorders is that patients become aware of a known medical illness, fixate on it, and eventually develop the belief they have acquired an amplified form of the disorder. This can be from the media, friends, or health agencies [14]. Another theory is that there is a buildup of dopamine in the striatum of the brain due to malfunctioning dopamine transporters. This theory is supported by some patients responding to treatment with dopamine antagonists [1,15]. The patient, in this case, has continued to experience the delusions while using a dopamine antagonist, namely Zyprexa, but with continued delusions.

## Conclusions

For the reasons mentioned above, we believe this 70-year-old woman has been experiencing primary delusional parasitosis. She has continued to present to many different specialists with her complaint and is adamant about the presence of the parasites, even when told they are not there. Due to the very low prevalence of this disorder, we believe it is valuable to document it in order to motivate research to understand and treat this phenomenon.

We further believe the patient is receiving the best care possible given her current condition. She is in a free-standing psychiatric hospital where psychotherapy is given priority, and the patient can develop trusting relationships with her providers which will aid in psychotherapy. Additionally, the patient’s Zyprexa regimen has not been controlling her delusions, but there is room for a dosage increase.
